# Supplementary material for: Genomic Analysis of Shiga Toxin-Producing E. coli O157 Cattle and Clinical Isolates from Alberta, Canada
Source: Toxins (Basel). 2022 Aug 31;14(9):603. doi: 10.3390/toxins14090603 (PMC9505746; doi:10.3390/toxins14090603)
Supplement: Supplementary file 1 [file toxins-14-00603-s001.zip › Table S5.pdf]

Table S5: Predicted plasmids replicons in cattle and clinical isolates

Plasmid replicon	Total number	Clinical	Cattle	Size/bp	Accession number
Col(BS512)	1		1	233	010656
Col(MG828)	3	2	1	262	008486
Col440I	2	2		112	CP023920.1
ColpVC	1	1		193	JX133088
ColRNAI	2		2	103	DQ298019
IncA/C2	1		1	417	JN157804
IncB/O/K/Z	2	2		156	GU256641
IncFIA	16	6	10	385	AP001918
IncFIA(HI1)	1		1	388	AF250878
IncFIB(AP001918)	242	126	116	682	AP001918
IncFIB(K)	1		1	560	JN233704
IncFIC(FII)	6		6	499	AP001918
IncFII	245	134	111	261	AY458016
IncFII(pCoo)	1	1		263	CR942285
IncFII(pHN7A8)	7	4	3	260	JN232517
IncHI2	1		1	327	BX664015
IncHI2A	1		1	63	BX664015
IncI	7		7	137	AP011954
IncI1	16	9	7	142	AP005147
IncI2	8	5	3	316	AP002527
IncN	1	1		514	AY046276
IncX1	2	1	1	374	EU370913
IncX4	4		4	712	FN543504
IncY	4		4	765	K02380
p0111	1	1		885	AP010962
pEC4115	13	12	1	706	011351
pXuzhou21	1	1		720	CP001927
